# Supplementary material for: Early life height and weight production functions with endogenous energy and protein inputs
Source: Econ Hum Biol. 2016 Sep;22:65–81. doi: 10.1016/j.ehb.2016.03.002 (PMC5001437; doi:10.1016/j.ehb.2016.03.002)
Supplement: Supplementary file 1 [file mmc1.docx]

**Data Appendix**

**1. Imputation of missing protein and non-protein intakes in Guatemala**

The data for Guatemala included information on protein and non-protein intakes calculated from home consumption (based on the periodic home dietary 24- and 72-hour recall) and, separately, from supplement consumption (recorded daily at the feeding center), where the supplement was either Atole or Fresco. Supplements were prepared under careful oversight and intakes carefully measured (e.g., monitoring any amounts not consumed after a child was done) so their nutrient contents are well measured. We constructed total protein and non-protein intakes adding the intakes from both home and supplement consumption. In principle, intake data are available for children at ages: 3, 6, 9, 12, 15, 18, 21, 24, 30, 36, 42, 48, 60, 72 and 84 months. As described in the text, average daily intakes from home consumption were calculated for a given period (e.g., 3-6 months) by averaging the intakes at the two endpoints and then total intakes from home consumption were calculated by multiplying this daily average by the exact number of days between measurements. Supplement intakes were measured on a daily basis and were thus summed over the period. Last, intakes from both sources were totaled.

For some children at some ages, however, these total intakes are unavailable (either because an intake measure from home diet or from supplement was missing). We estimated flexible individual-level fixed-effects models for protein and non-protein intakes, separately, and used these models to impute some of the missing intakes. The model included as covariates age and year-of-birth dummies as shown in Table 1. Although our main analyses in the paper are from 6-24 months, the imputation model uses observation on all available ages (up to age 84 months) to improve the estimation of the individual effect.

Using the estimated individual fixed-effect models, we predicted intake values for a missing observation only when it was adjacent in time period to an observed intake, but we did not impute intakes if it was more than one measurement period away from an observed intake. For example, if there was missing information on intakes for a child at both ages 3, 6 and 9 months of age, but there was information at age 12, we predicted intakes at age 9, but not age 6, which remained as a missing observationa nd consequently the observation for that age for that child was not included in the estimation. In the previous example, if there was a measurement at age 3 months, we used the model to predict intake at age 6 months.

Appendix Table A1: Fixed-effect models for Protein and Non-Protein intakes in Guatemala

|  | (1) |  | (2) |  |
| --- | --- | --- | --- | --- |
|  | Protein |  | Non-Protein | |
| Age in months |  |  |  |  |
| 3 months | omitted |  | omitted |  |
| 6 months | 12.24 | (4.938) | 76.11 | (4.261) |
| 9 months | 26.79 | (10.311) | 197.9 | (10.575) |
| 12 months | 41.81 | (15.041) | 324.6 | (16.208) |
| 15 months | 64.60 | (22.689) | 520.5 | (25.373) |
| 18 months | 81.40 | (26.904) | 625.8 | (28.712) |
| 21 months | 93.36 | (28.749) | 702.6 | (30.032) |
| 24 months | 107.2 | (30.375) | 787.8 | (30.971) |
| 30 months | 128.1 | (31.709) | 891.9 | (30.643) |
| 36 months | 142.3 | (30.461) | 984.4 | (29.254) |
| 42 months | 148.7 | (28.404) | 1009.9 | (26.781) |
| 48 months | 158.4 | (26.808) | 1092.9 | (25.674) |
| 60 months | 174.3 | (24.070) | 1179.4 | (22.614) |
| 72 months | 193.9 | (21.808) | 1287.1 | (20.092) |
| 84 months | 214.3 | (20.783) | 1432.2 | (19.277) |
| Birth year (=1) |  |  |  |  |
| 1968 | omitted |  | omitted |  |
| 1969 | -5.406 | (-1.063) | -63.16 | (-1.723) |
| 1970 | -12.96 | (-2.289) | -138.6 | (-3.397) |
| 1971 | -15.80 | (-2.433) | -189.8 | (-4.056) |
| 1972 | -32.21 | (-4.257) | -273.3 | (-5.013) |
| 1973 | -37.78 | (-4.324) | -276.6 | (-4.394) |
| 1974 | -45.70 | (-4.599) | -289.8 | (-4.049) |
| 1975 | -49.62 | (-4.436) | -285.8 | (-3.547) |
| 1976 | -48.10 | (-3.874) | -257.1 | (-2.874) |
| 1977 | -54.00 | (-4.027) | -274.6 | (-2.842) |
| Constant | 31.42 | (4.382) | 225.2 | (4.360) |
| Observations | 10117 |  | 10117 |  |

*t* statistics in parentheses

**2. Diarrhea Models**

The data from Guatemala includes detailed information on diarrhea including the number of days with diarrhea symptoms for each 15-day period during the first 24 months.^[[1]](#footnote-1)^ However, when one of these interviews was not conducted, the information on the number of days with diarrhea symptoms for that period are missing. To obtain an estimate of the total number of days with diarrhea for each growth period for the Guatemala analyses, we multiplied the average days of diarrhea of the observed period, with the length of the growth period, thereby assuming a similar incidence of diarrhea for the observed and unobserved days, as described in the text.

In the case of the Philippines, however, the data available for diarrhea only cover the seven days before each anthropometric measurement. To obtain a prediction of days with diarrhea for the entire 2-month growth period, we estimated count models using data from Guatemala, mimicking the information available in the Philippines, and then used those estimates to predict the number of days with diarrhea for each growth period in the Philippines. We took advantage detailed data from Guatemala with information on all the 15-days interviews to construct 2-month intervals for this estimation.

In specific, we estimated a count model to predict the total number of days with diarrhea for each two-month period; we used two-month periods to correspond directly to the data collection in the Philippines, which was every two months for each child. Since the prevalence of diarrhea varies with the age of the child, we estimated different models for each 2-month growth period, i.e., 0-2, 2-4, 4-6, etc. until 22-24 months of age.

There are a variety of different distributional functions that can be used for the count process when estimating a count model. With the aim of finding an accurate prediction model, we considered four different commonly used distributions: Poisson, Negative Binomial, Zero-Inflated Poisson and Zero-Inflated Negative Binomial model. All models were estimated using maximum likelihood.

The Poisson distribution implies that the probability of observing $y$ days of diarrhea in a given two-month period is:

$$Pr\left[ Y=y \right]=\frac{exp\left( \mu_{i} \right){\mu_{i}}^{y}}{y!}$$

where $\mu_{i}=exp(x_{i}^{'}\beta)$ and $x_{i}$ are covariates. We used a limited set of covariates for each count model: the number of days with diarrhea in the last 15 days before a child is age *m* months ($x_{m,15})$; the average number of days of diarrhea of children of the same age in the same village over the same period; and indicators of the sex and birth order of the child. These covariates were chosen because of their availability across both samples, allowing us to apply the estimated coefficients from the model estimated with Guatemalan data to the Philippine data.

Since the objective of this estimation was to maximize the predictive power of the model, we evaluated different specifications of the covariates, always maintaining the basic variables described above. The additional covariates we included were variations of $x_{m,15}$; for instance, in the count model of the number of days with diarrhea during the growth period from 6 to 8 months of age, we can include not only the number of days with diarrhea 15 days before the child was age 8 months $x_{8,15}$, but also the number of days with diarrhea in the last 15 days previous to when the child was 6 months of age $x_{6,15}$ or the number of days with diarrhea in the last 15 days previous to measurement when the child was 10 months of age $x_{10,15}$ and so on.

To evaluate which model had the best fit we calculated the Bayesian information criteria (BIC) and an R^2^ using the prediction of each model. To select the best specification and distribution we proceeded as follows. We compared (on the BIC and R^2^) the specifications using the four potential distributions: Poisson, Negative Binomial count model, a Zero-Inflated Poisson model and a Zero-Inflated Negative Binomial model. At the same time, we calculated several specifications using different sets of the series of $\left( x_{m,15} \right)$ from month 0 to month 24. Our results indicate first, that to predict the number of days with diarrhea in any growth period, all 15-day diarrhea measures (including both the “past” and the “future”) should be included. Second, in terms of the BIC criteria, the Negative Binomial model outperformed the other distributions, but in terms of the R^2^, the Poisson distribution was best. Then, to select the model that had the best fit for the Philippines between these two, we predicted the days without diarrhea using the Poisson and Negative binomial models, and correlated the predicted values with the actual information observed for the Philippines^[[2]](#footnote-3)^. We found that the Poisson model had the highest correlation. Therefore we selected the Poisson distribution to predict the number of days without diarrhea for all two-month periods in the Philippines.

Following are the 12 models we used to predict days with diarrhea for the 12 growth periods in the Philippines.

Appendix Table A2: Estimation of Count Models of Number of Days with Diarrhea

|  | (1) |  |
| --- | --- | --- |
| Number of Days with Diarrhea from age 0 mo to age 2 mo | | |
| Days with diarrhea during 15 days previous to age: |  |  |
| 2 months | 0.287 | (29.761) |
| 4 months | 0.107 | (8.797) |
| 6 mo old | -0.0470 | (-3.629) |
| 8 mo old | -0.0241 | (-1.554) |
| 10 mo old | 0.0605 | (4.229) |
| 12 mo old | -0.0490 | (-3.921) |
| 14 mo old | -0.0623 | (-4.363) |
| 16 mo old | 0.0439 | (2.243) |
| 18 mo old | 0.0743 | (4.526) |
| 20 mo old | -0.0896 | (-4.178) |
| 22 mo old | 0.0343 | (1.903) |
| 24 mo old | -0.0695 | (-2.509) |
| Average days of diarrhea for children of same age, in same village, at same time | -0.558 | (-3.397) |
| Female (=1) | 0.123 | (0.976) |
| First born (=1) | -0.475 | (-2.590) |
| Second born (=1) | -2.391 | (-8.681) |
| Third born (=1) | 0.112 | (0.560) |
| Constant | 0.575 | (3.213) |
| Observations | 163 |  |

*t* statistics in parentheses

Count Model assuming Poisson Distribution. Maximum Likelihood Estimation.

Appendix Table A3: Number of Days with Diarrhea from age 2 mo to age 4 mo

|  | (1) | |  | |  |
| --- | --- | --- | --- | --- | --- |
| Number of Days with Diarrhea from age 2 mo to age 4 mo | | | | |  |
| Days with diarrhea during 15 days previous to age: | |  | |  | |
| 2 months | | 0.116 | | (18.447) | |
| 4 months | | 0.144 | | (21.101) | |
| 6 mo old | | 0.0491 | | (6.149) | |
| 8 mo old | | 0.0293 | | (3.606) | |
| 10 mo old | | 0.0686 | | (7.317) | |
| 12 mo old | | 0.00462 | | (0.581) | |
| 14 mo old | | 0.0545 | | (8.010) | |
| 16 mo old | | 0.00447 | | (0.406) | |
| 18 mo old | | -0.0198 | | (-2.004) | |
| 20 mo old | | -0.0507 | | (-4.396) | |
| 22 mo old | | -0.108 | | (-7.217) | |
| 24 mo old | | 0.0808 | | (7.101) | |
| Average days of diarrhea for children of same age, in same village, at same time | | 0.677 | | (6.731) | |
| Female (=1) | | 0.264 | | (3.120) | |
| First born (=1) | | -0.795 | | (-3.837) | |
| Second born (=1) | | 0.0881 | | (0.882) | |
| Third born (=1) | | 0.375 | | (2.477) | |
| Constant | | -0.736 | | (-4.335) | |
| Observations | 224 | |  | |  |

*t* statistics in parentheses

Count Model assuming Poisson Distribution. Maximum Likelihood Estimation.

Appendix Table A4: Number of Days with Diarrhea from age 4 mo to age 6 mo

|  | (1) |  |
| --- | --- | --- |
| Number of Days with Diarrhea from age 4 mo to age 6 mo | | |
| Days with diarrhea during 15 days previous to age: |  |  |
| 2 months | 0.0293 | (4.217) |
| 4 months | 0.0913 | (14.936) |
| 6 mo old | 0.147 | (24.269) |
| 8 mo old | -0.0139 | (-1.806) |
| 10 mo old | 0.0494 | (7.200) |
| 12 mo old | 0.0203 | (3.001) |
| 14 mo old | 0.0612 | (9.944) |
| 16 mo old | -0.00174 | (-0.192) |
| 18 mo old | 0.0299 | (5.003) |
| 20 mo old | 0.0680 | (8.076) |
| 22 mo old | 0.0113 | (1.393) |
| 24 mo old | -0.0320 | (-2.896) |
| Average days of diarrhea for children of same age, in same village, at same time | 0.684 | (8.752) |
| Female (=1) | 0.104 | (1.512) |
| First born (=1) | -0.419 | (-3.139) |
| Second born (=1) | 0.236 | (2.619) |
| Third born (=1) | 0.153 | (1.268) |
| Constant | -0.730 | (-4.710) |
| Observations | 230 |  |

*t* statistics in parentheses

Count Model assuming Poisson Distribution. Maximum Likelihood Estimation.

Appendix Table A5: Number of Days with Diarrhea from age 6 mo to age 8 mo

|  | (1) |  |
| --- | --- | --- |
| Number of Days with Diarrhea from age 6 mo to age 8 mo | | |
| Days with diarrhea during 15 days previous to age: |  |  |
| 2 months | -0.00964 | (-1.283) |
| 4 months | -0.0136 | (-2.092) |
| 6 mo old | 0.0708 | (14.529) |
| 8 mo old | 0.123 | (21.737) |
| 10 mo old | -0.0151 | (-2.539) |
| 12 mo old | 0.0123 | (1.992) |
| 14 mo old | -0.0135 | (-2.045) |
| 16 mo old | 0.0464 | (5.867) |
| 18 mo old | 0.00665 | (1.193) |
| 20 mo old | 0.0281 | (3.608) |
| 22 mo old | 0.0417 | (5.826) |
| 24 mo old | 0.0337 | (3.732) |
| Average days of diarrhea for children of same age, in same village, at same time | 0.229 | (2.763) |
| Female (=1) | -0.294 | (-4.883) |
| First born (=1) | 0.296 | (3.002) |
| Second born (=1) | -0.138 | (-1.779) |
| Third born (=1) | 0.165 | (1.756) |
| Constant | 0.853 | (5.756) |
| Observations | 222 |  |

*t* statistics in parentheses

Count Model assuming Poisson Distribution. Maximum Likelihood Estimation.

Appendix Table A6: Number of Days with Diarrhea from age 8 mo to age 10 mo

| (1)  Number of Days with Diarrhea from age 8 mo to age 10 mo | | |
| --- | --- | --- |
| Days with diarrhea during 15 days previous to age: |  |  |
| 2 months | -0.00167 | (-0.255) |
| 4 months | 0.00166 | (0.231) |
| 6 mo old | 0.0137 | (2.459) |
| 8 mo old | 0.0289 | (5.435) |
| 10 mo old | 0.111 | (25.650) |
| 12 mo old | 0.0000986 | (0.019) |
| 14 mo old | 0.0444 | (8.275) |
| 16 mo old | 0.0232 | (3.595) |
| 18 mo old | 0.0158 | (2.983) |
| 20 mo old | -0.00989 | (-1.270) |
| 22 mo old | 0.0345 | (5.328) |
| 24 mo old | -0.000590 | (-0.069) |
| Average days of diarrhea for children of same age, in same village, at same time | 0.410 | (6.321) |
| Female (=1) | -0.196 | (-4.140) |
| First born (=1) | 0.134 | (1.642) |
| Second born (=1) | -0.139 | (-1.839) |
| Third born (=1) | 0.0317 | (0.375) |
| Constant | 0.600 | (3.727) |
| Observations | 222 |  |

*t* statistics in parentheses

Count Model assuming Poisson Distribution. Maximum Likelihood Estimation.

Appendix Table A7: Number of Days with Diarrhea from age 10 mo to age 12 mo

| (1)  Number of Days with Diarrhea from age 10 mo to age 12 mo | | |
| --- | --- | --- |
| Days with diarrhea during 15 days previous to age: |  |  |
| 2 months | 0.0275 | (5.138) |
| 4 months | -0.00910 | (-1.216) |
| 6 mo old | -0.0183 | (-2.968) |
| 8 mo old | 0.0121 | (2.189) |
| 10 mo old | 0.0508 | (10.795) |
| 12 mo old | 0.0926 | (20.325) |
| 14 mo old | 0.0332 | (6.712) |
| 16 mo old | 0.0151 | (2.168) |
| 18 mo old | 0.00732 | (1.286) |
| 20 mo old | -0.0168 | (-2.087) |
| 22 mo old | 0.0599 | (8.622) |
| 24 mo old | 0.0151 | (1.776) |
| Average days of diarrhea for children of same age, in same village, at same time | 0.487 | (8.003) |
| Female (=1) | -0.00107 | (-0.023) |
| First born (=1) | -0.0393 | (-0.443) |
| Second born (=1) | 0.734 | (11.764) |
| Third born (=1) | -0.730 | (-5.507) |
| Constant | 0.392 | (2.838) |
| Observations | 232 |  |

*t* statistics in parentheses

Count Model assuming Poisson Distribution. Maximum Likelihood Estimation.

Appendix Table A8: Number of Days with Diarrhea from age 12 mo to age 14 mo

| (1)  Number of Days with Diarrhea from age 12 mo to age 14 mo | | |
| --- | --- | --- |
| Days with diarrhea during 15 days previous to age: |  |  |
| 2 months | 0.0448 | (8.110) |
| 4 months | -0.0419 | (-4.520) |
| 6 mo old | 0.0247 | (4.084) |
| 8 mo old | -0.0192 | (-2.854) |
| 10 mo old | 0.0196 | (3.386) |
| 12 mo old | 0.0382 | (7.374) |
| 14 mo old | 0.151 | (28.242) |
| 16 mo old | 0.00744 | (0.840) |
| 18 mo old | 0.0130 | (2.105) |
| 20 mo old | -0.0181 | (-1.839) |
| 22 mo old | 0.0284 | (3.824) |
| 24 mo old | 0.0517 | (5.846) |
| Average days of diarrhea for children of same age, in same village, at same time | 0.0626 | (0.922) |
| Female (=1) | 0.121 | (2.288) |
| First born (=1) | 0.316 | (3.767) |
| Second born (=1) | -0.124 | (-1.455) |
| Third born (=1) | 0.0627 | (0.569) |
| Constant | 0.982 | (6.751) |
| Observations | 225 |  |

*t* statistics in parentheses

Count Model assuming Poisson Distribution. Maximum Likelihood Estimation.

Appendix Table A9: Number of Days with Diarrhea from age 14 mo to age 16 mo

| (1)  Number of Days with Diarrhea from age 14 mo to age 16 mo | | |
| --- | --- | --- |
| Days with diarrhea during 15 days previous to age: |  |  |
| 2 months | 0.0271 | (4.449) |
| 4 months | -0.0429 | (-5.214) |
| 6 mo old | 0.0357 | (6.074) |
| 8 mo old | 0.0566 | (9.995) |
| 10 mo old | -0.0161 | (-2.649) |
| 12 mo old | -0.00774 | (-1.258) |
| 14 mo old | 0.0569 | (9.994) |
| 16 mo old | 0.133 | (21.063) |
| 18 mo old | 0.0108 | (1.904) |
| 20 mo old | 0.0281 | (4.062) |
| 22 mo old | 0.0555 | (8.296) |
| 24 mo old | -0.00892 | (-1.032) |
| Average days of diarrhea for children of same age, in same village, at same time | -0.0138 | (-0.210) |
| Female (=1) | -0.0117 | (-0.208) |
| First born (=1) | -0.468 | (-4.175) |
| Second born (=1) | -0.233 | (-2.903) |
| Third born (=1) | -0.0504 | (-0.495) |
| Constant | 1.201 | (9.461) |
| Observations | 231 |  |

*t* statistics in parentheses

Count Model assuming Poisson Distribution. Maximum Likelihood Estimation.

Appendix Table A10: Number of Days with Diarrhea from age 16 mo to age 18 mo

| (1)  Number of Days with Diarrhea from age 16 mo to age 18 mo | | |
| --- | --- | --- |
| Days with diarrhea during 15 days previous to age: |  |  |
| 2 months | 0.00473 | (0.595) |
| 4 months | -0.0179 | (-1.853) |
| 6 mo old | 0.00613 | (0.984) |
| 8 mo old | 0.0733 | (10.689) |
| 10 mo old | -0.0577 | (-8.005) |
| 12 mo old | 0.0109 | (1.704) |
| 14 mo old | 0.0143 | (1.972) |
| 16 mo old | 0.106 | (15.217) |
| 18 mo old | 0.116 | (19.520) |
| 20 mo old | -0.00579 | (-0.656) |
| 22 mo old | 0.0252 | (2.870) |
| 24 mo old | 0.0325 | (3.633) |
| Average days of diarrhea for children of same age, in same village, at same time | 0.504 | (6.553) |
| Female (=1) | 0.152 | (2.567) |
| First born (=1) | -0.129 | (-1.383) |
| Second born (=1) | -0.102 | (-1.265) |
| Third born (=1) | -0.189 | (-1.640) |
| Constant | 0.0817 | (0.478) |
| Observations | 228 |  |

*t* statistics in parentheses

Count Model assuming Poisson Distribution. Maximum Likelihood Estimation.

Appendix Table A11: Number of Days with Diarrhea from age 18 mo to age 20 mo

| (1)  Number of Days with Diarrhea from age 18 mo to age 20 mo | | |
| --- | --- | --- |
| Days with diarrhea during 15 days previous to age: |  |  |
| 2 months | -0.0461 | (-5.830) |
| 4 months | 0.00105 | (0.126) |
| 6 mo old | 0.00917 | (1.306) |
| 8 mo old | 0.0310 | (4.103) |
| 10 mo old | 0.00640 | (1.007) |
| 12 mo old | -0.0314 | (-4.250) |
| 14 mo old | 0.0814 | (13.091) |
| 16 mo old | -0.0250 | (-3.031) |
| 18 mo old | 0.0907 | (16.077) |
| 20 mo old | 0.159 | (25.675) |
| 22 mo old | 0.0171 | (2.203) |
| 24 mo old | 0.0307 | (3.986) |
| Average days of diarrhea for children of same age, in same village, at same time | -0.0692 | (-0.722) |
| Female (=1) | -0.0129 | (-0.226) |
| First born (=1) | 0.350 | (3.806) |
| Second born (=1) | 0.0976 | (1.110) |
| Third born (=1) | 0.208 | (2.267) |
| Constant | 1.025 | (7.206) |
| Observations | 231 |  |

*t* statistics in parentheses

Count Model assuming Poisson Distribution. Maximum Likelihood Estimation.

Appendix Table A12 Number of Days with Diarrhea from age 20 mo to age 22 mo

| (1)  Number of Days with Diarrhea from age 20 mo to age 22 mo | | |
| --- | --- | --- |
| Days with diarrhea during 15 days previous to age: |  |  |
| 2 months | 0.0186 | (2.305) |
| 4 months | -0.0185 | (-2.029) |
| 6 mo old | 0.0374 | (5.732) |
| 8 mo old | 0.0231 | (3.154) |
| 10 mo old | 0.0102 | (1.557) |
| 12 mo old | 0.0284 | (3.896) |
| 14 mo old | 0.0290 | (4.083) |
| 16 mo old | 0.0130 | (1.510) |
| 18 mo old | 0.0119 | (1.834) |
| 20 mo old | 0.0943 | (13.018) |
| 22 mo old | 0.150 | (22.769) |
| 24 mo old | 0.0342 | (4.147) |
| Average days of diarrhea for children of same age, in same village, at same time | 0.271 | (3.697) |
| Female (=1) | 0.0641 | (1.008) |
| First born (=1) | -0.417 | (-2.958) |
| Second born (=1) | 0.516 | (5.429) |
| Third born (=1) | -0.234 | (-2.297) |
| Constant | 0.334 | (2.956) |
| Observations | 223 |  |

*t* statistics in parentheses

Count Model assuming Poisson Distribution. Maximum Likelihood Estimation.

Appendix Table A13: Number of Days with Diarrhea from age 22 mo to age 24 mo

| (1)  Number of Days with Diarrhea from age 22 mo to age 24 mo | | |
| --- | --- | --- |
| Days with diarrhea during 15 days previous to age: |  |  |
| 2 months | 0.0363 | (3.757) |
| 4 months | -0.0254 | (-2.108) |
| 6 mo old | -0.0176 | (-1.920) |
| 8 mo old | 0.0564 | (6.174) |
| 10 mo old | -0.0124 | (-1.453) |
| 12 mo old | -0.0105 | (-1.093) |
| 14 mo old | -0.00228 | (-0.288) |
| 16 mo old | -0.0120 | (-1.159) |
| 18 mo old | 0.0441 | (5.108) |
| 20 mo old | 0.0167 | (2.038) |
| 22 mo old | 0.120 | (13.621) |
| 24 mo old | 0.187 | (26.108) |
| Average days of diarrhea for children of same age, in same village, at same time | 0.0989 | (1.057) |
| Female (=1) | 0.138 | (1.798) |
| First born (=1) | -0.399 | (-2.474) |
| Second born (=1) | -0.418 | (-3.476) |
| Third born (=1) | -0.861 | (-5.692) |
| Constant | 0.524 | (4.370) |
| Observations | 232 |  |

*t* statistics in parentheses

Count Model assuming Poisson Distribution. Maximum Likelihood Estimation.

**3. Food prices used in the instrument sets**

*Guatemala*

For Guatemala, we use lagged annual prices of eggs, chicken, pork, beef, dry beans, corn, and rice, obtained from the FAO through its website FAOSTAT. All prices are available over the eight-year study period (and thus range from 1968 to 1976).

*Philippines*

For the Philippines, we use community-specific^[[3]](#footnote-4)^ prices collected as part of the broader study. Beginning in January 1983 (and ending in May 1986), enumerators visited two stores in each community, every other month, and collected prices (and quantity units) for a list of items. Not all items, however, were sold at each store at each visit. Consequently, there is not a complete set of prices for each item from each store (or even from each community in instances where no price was available from either store) in each measurement period.

1. Conversion to unit prices
   - For the same product, across different stores or visits, not all prices were collected in the same units (possible units included for example piece, hundred, pack, kg and chupa). Prices per unit also differed for goods for sale in different quantities.
   - All prices were converted to the value in Philippine Centavos for 100 grams of product.
2. Averaging, Outliers and Interpolation
   - When a unit price was available for both stores in a community at a given time, then the average of those prices was used for that community and time period. If only one unit price was available, then that price was used for that community and time period.
   - There were some examples of extreme values of unit prices, possibly due to incorrect unit conversions. A small percentage of these extreme values were dropped.
   - As the price surveys were administered during odd numbered months (January, March, etc.), prices for even numbered months were calculated as the average of two adjacent odd numbered months.
3. Our analyses use as instruments prices for dried fish, eggs, corn, and tomatoes. Although prices for many other food items also were collected (including chicken, beef, pork, fried fish, and cabbage), they were often missing. After interpolating for even months, the number of community-month observations on different prices are as follows, out of a maximum possible of 1353:

Food items used as instruments

- - Corn (N= 835)
  - Dried Fish (N=760)
  - Eggs (N=783)
  - Tomatoes (N=794)

Food items not used as instruments

- - C4 rice (N=497)
  - Wagwag rice (N=318)
  - Pork (N=262)
  - Beef (N=218)
  - Chicken (N=176)

All prices were inflated to year 2000 Philippine Centavos using monthly inflation rates. These were obtained from the website of Bangko Sentralng Pilipinas, the Central Bank of the Philippines: <http://www.bsp.gov.ph/dbank_reports/Prices_1_rpt.asp?frequency=Monthly&range_from=1980&range_to=2011&conversion=None> accessed on September 17^th^, 2014.

**4. Specifications and number of observations**

In this section, we examine whether the point estimates for the Philippines vary with the number of observations used; the driving factor behind the differing numbers of observations across specifications is the availability of food prices for the each particular set of instruments used, as described in the previous section. The number of observations ranges from approximately 7,000 to 15,000.

We present four figures. Figure 1 shows the protein coefficients and figure 2 the non-protein coefficients for the height production function. Figures 3 and 4 show the protein and non-protein coefficients, respectively, for the weight production function. We graph the point estimates (y-axis) against the number of observations (x-axis) for all specifications, dividing each set of results into two sub-panels. The left-side panel displays specifications with a CD < 3 and the right-side panel, with CD >3. The figures also indicate which specifications meet the 0.1 threshold for the p-value of the HJ as defined in the legend.

The graphs indicate that there does not appear to be any relationship between the point estimates and the number of observations. Nor does there appear to be evidence that they systematically vary according by the cut-off of 0.1 for the p-value of the HJ statistics.

Appendix Figure A1: Protein Coefficient in the Height Specification and Number of Observations


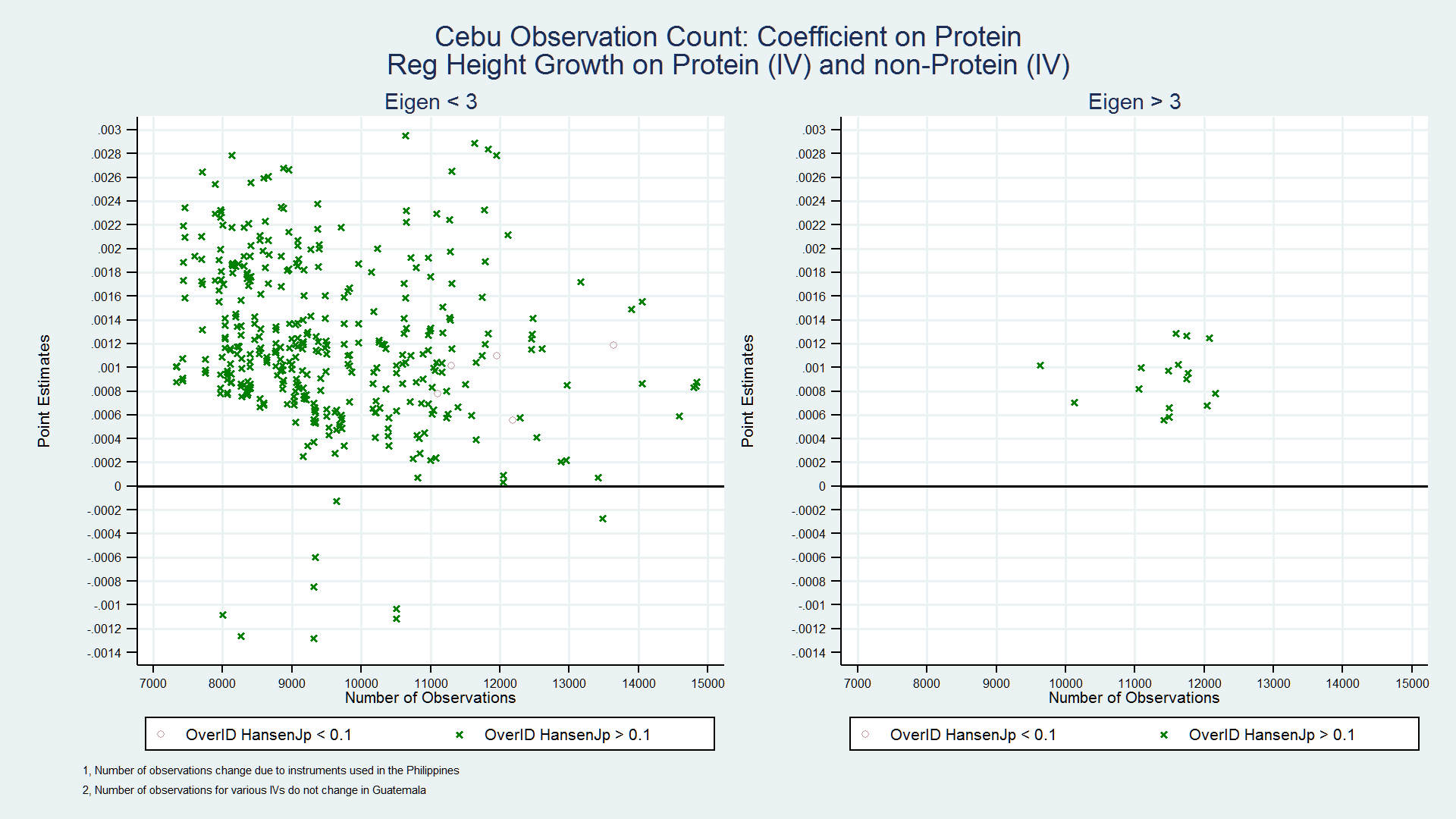


Appendix Figure A2: Non-Protein Coefficient in the Height Specification and Number of Observations


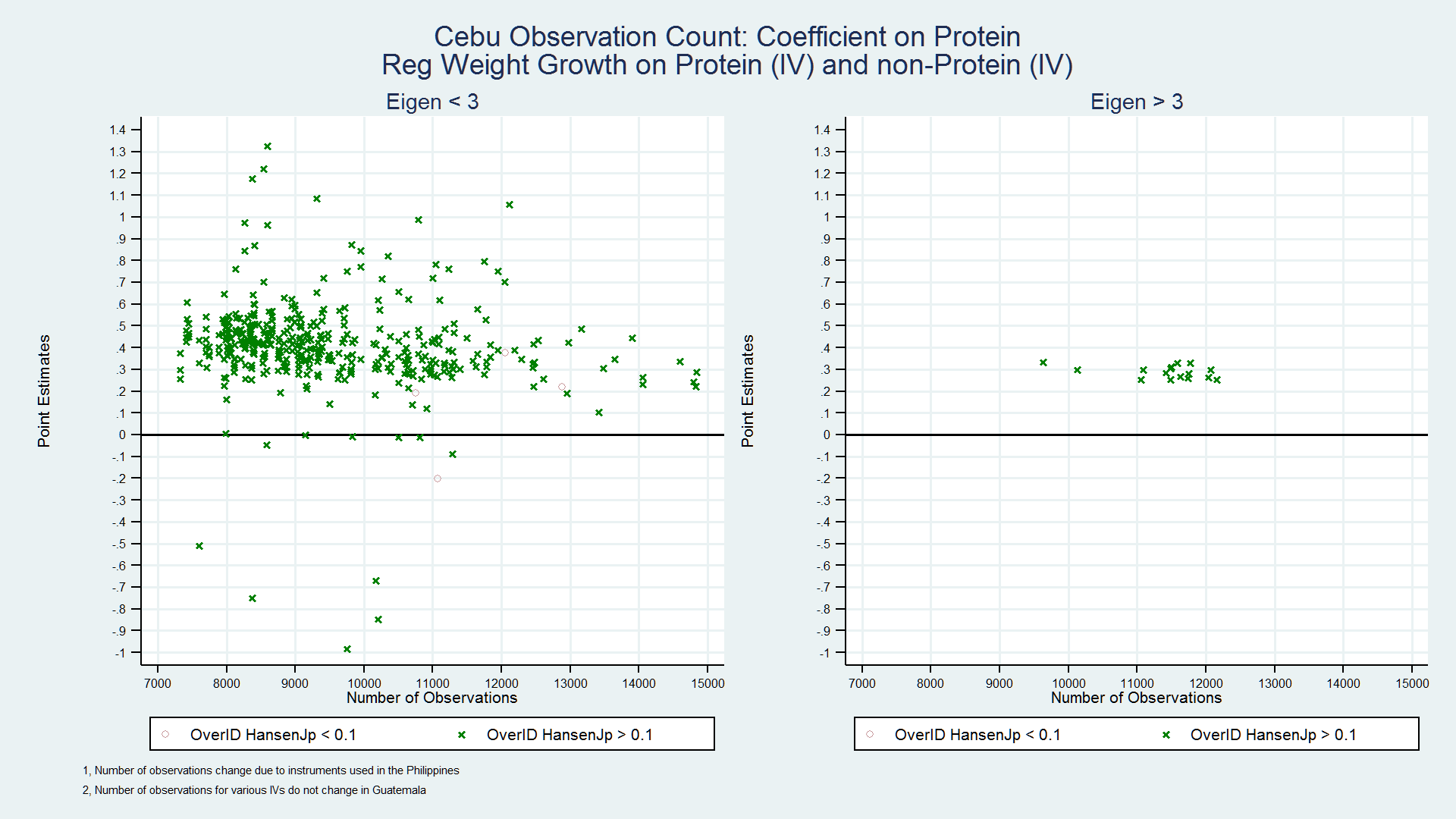


Appendix Figure A3: Protein Coefficient in the Weight Specification and Number of Observations


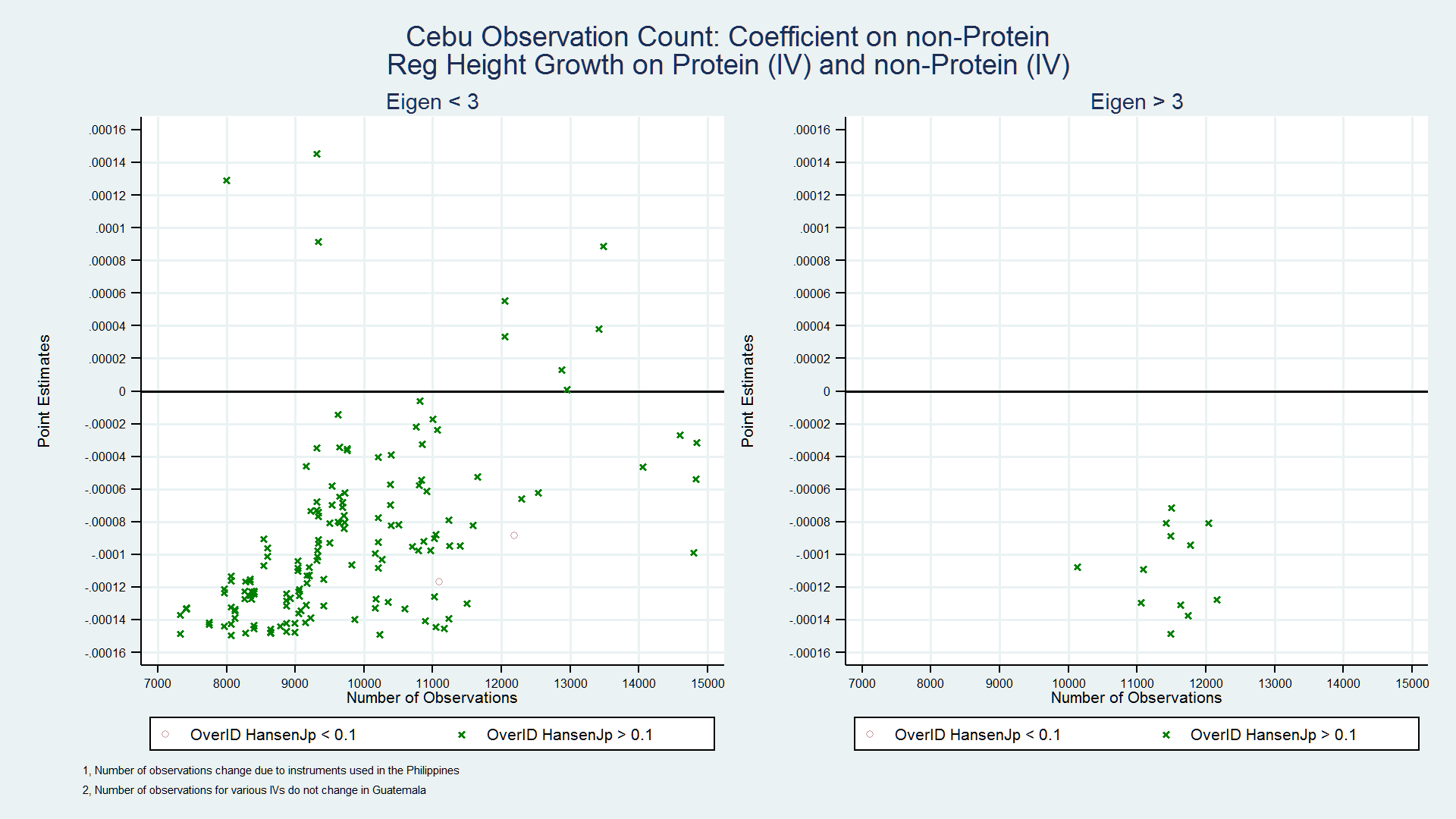


Appendix Figure A4: Non-Protein Coefficient in the Weight Specification and Number of Observations


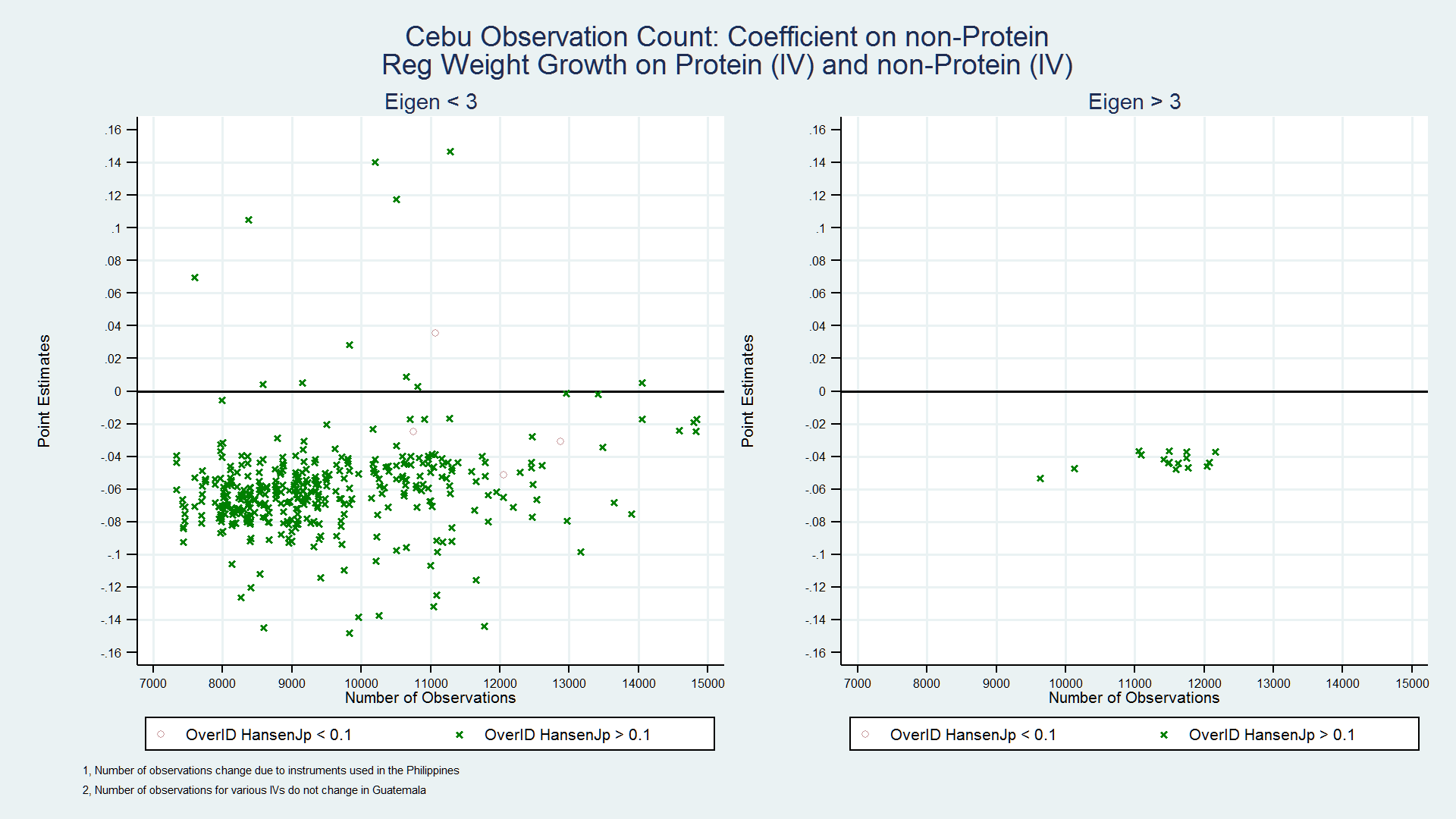


**5. Height and Weight production functions**

In this section, we present the set of Figures paralleling Figures 1 to 3 in the paper, but using a cutoff of 0.10 for the p-value of the Hansen-J test. Results are little changed from those with a cutoff of 0.05 discussed in the paper.

Figure A5: Total Energy Coefficient

Change in Height: Estimates $\lambda_{energy}^{h}$ from Equation (5) in the manuscript


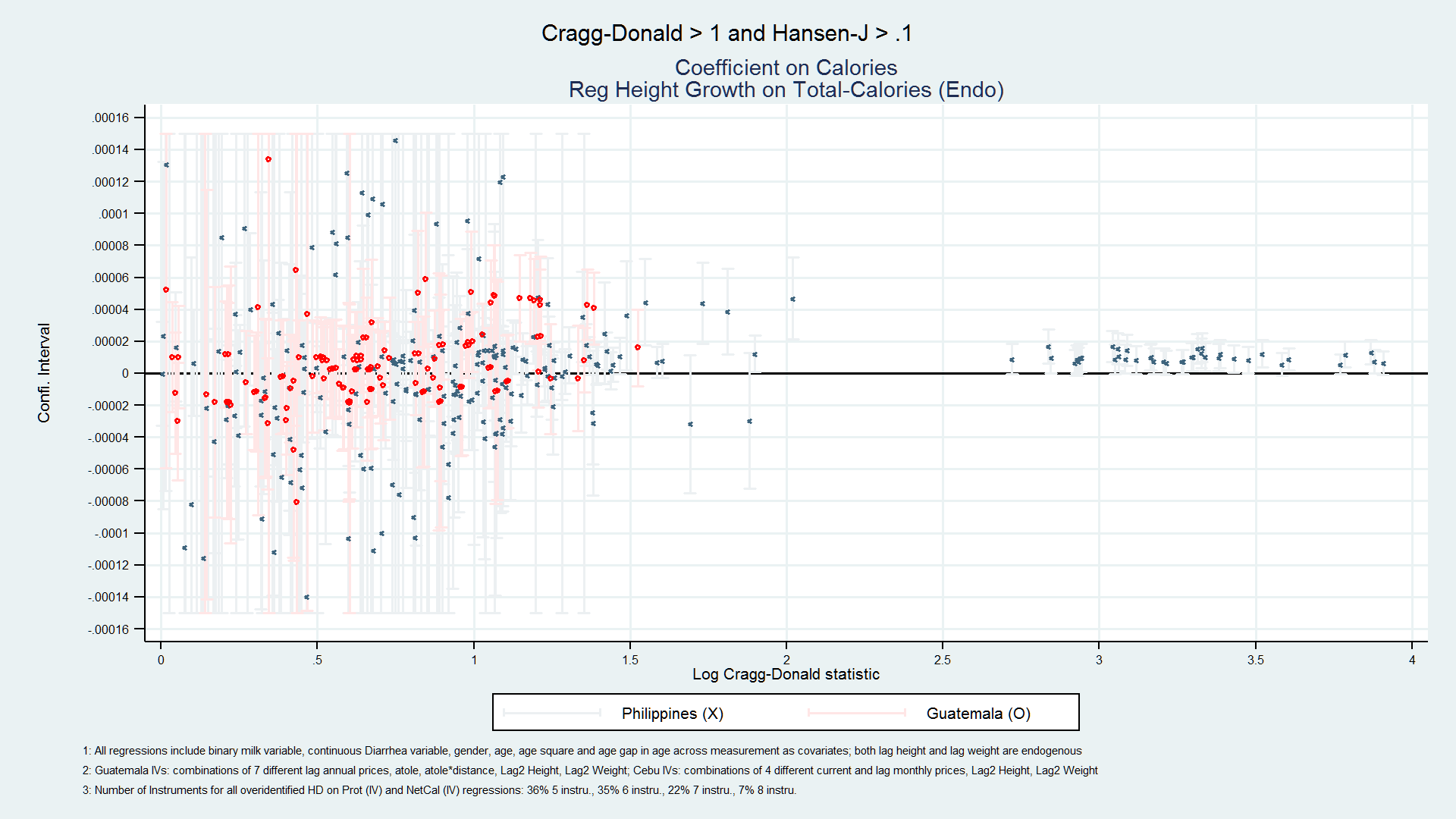


Change in Weight: Estimates $\lambda_{energy}^{w}$ Equation (5) in the manuscript


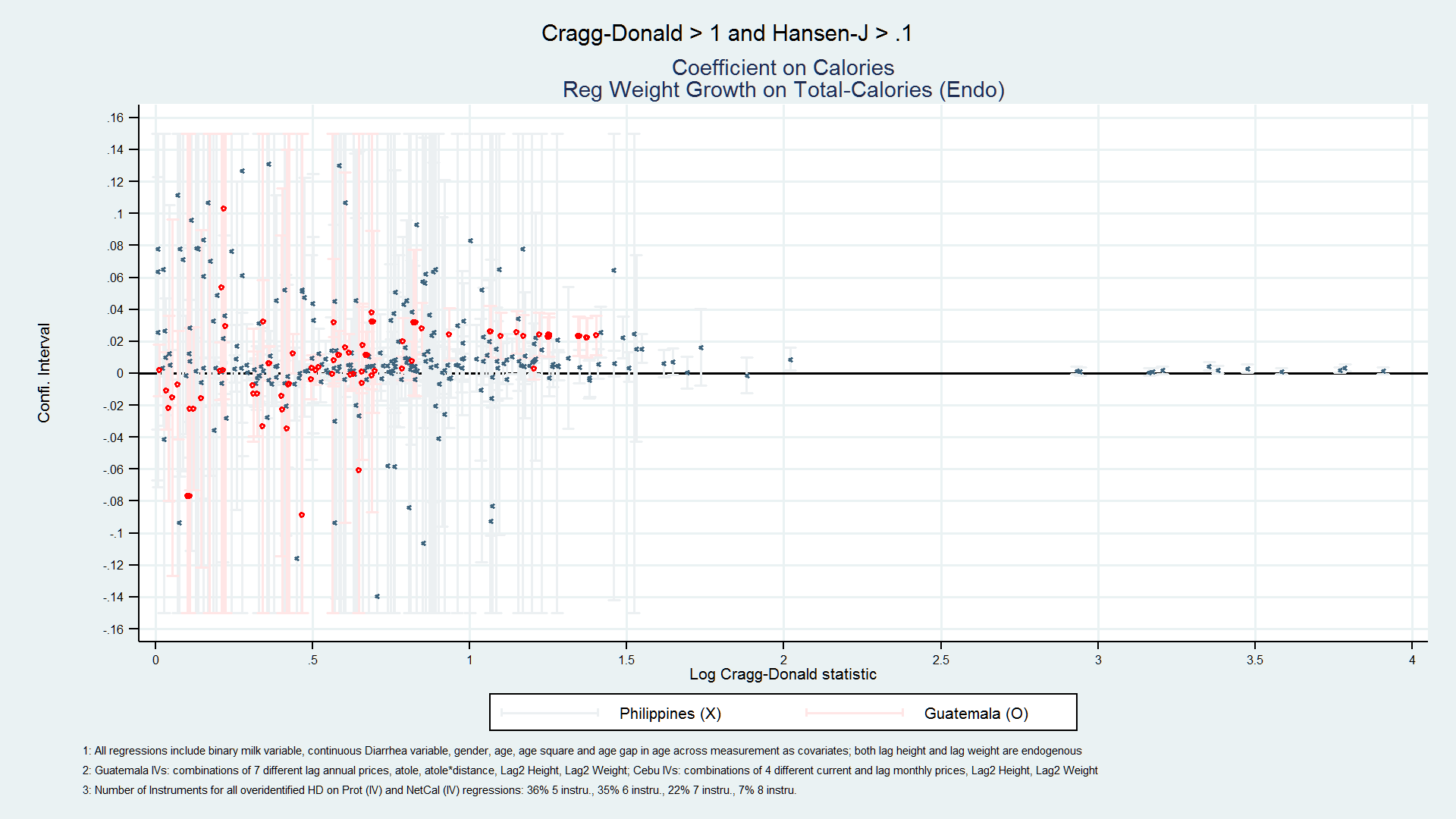


Figure A6: Protein Coefficient

Change in Height: Estimates $\lambda_{prot}^{h}$ from Equation (6) in the manuscript


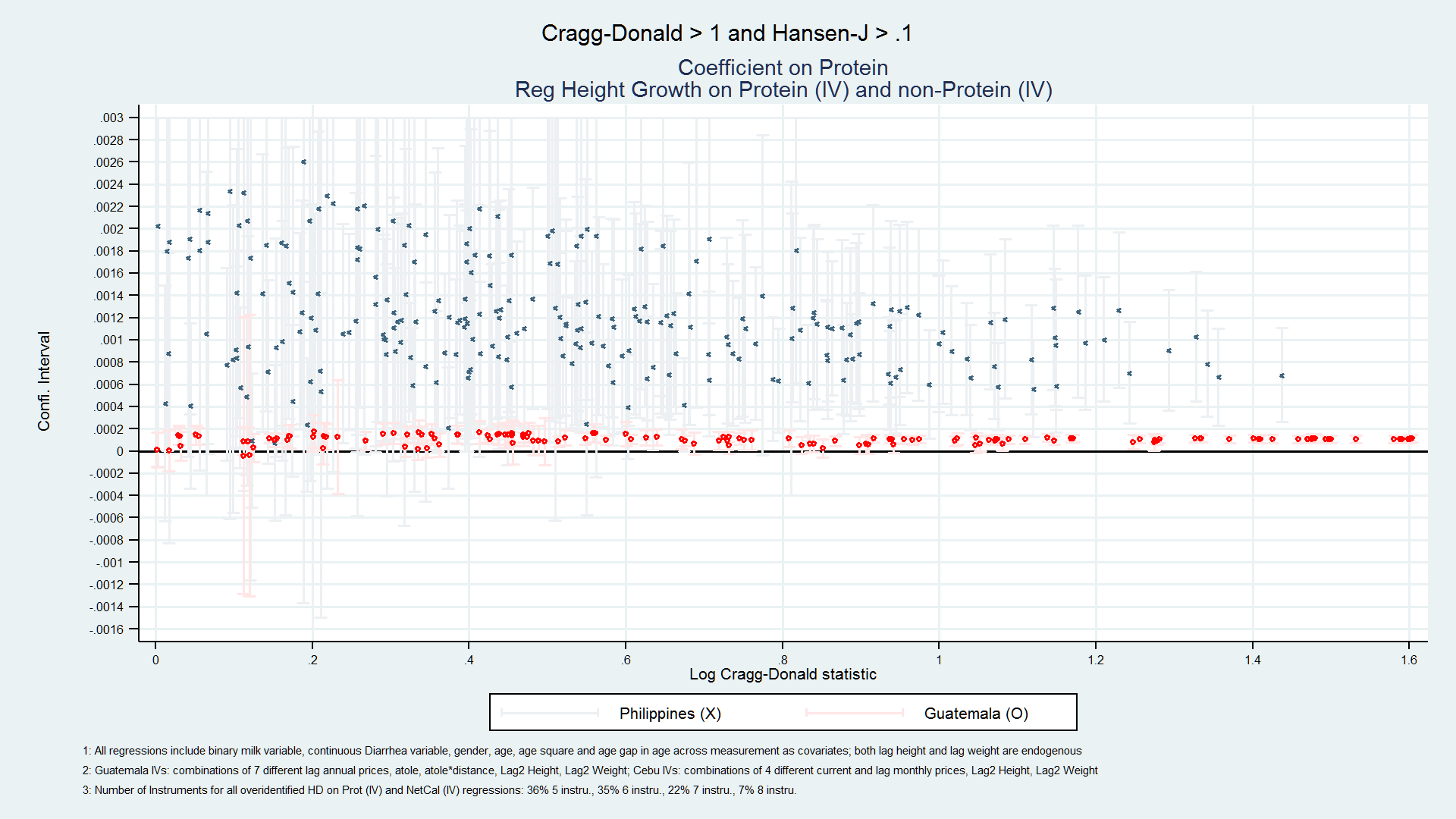


Change in Weight: Estimates $\lambda_{prot}^{w}$ from Equation (6) in the manuscript


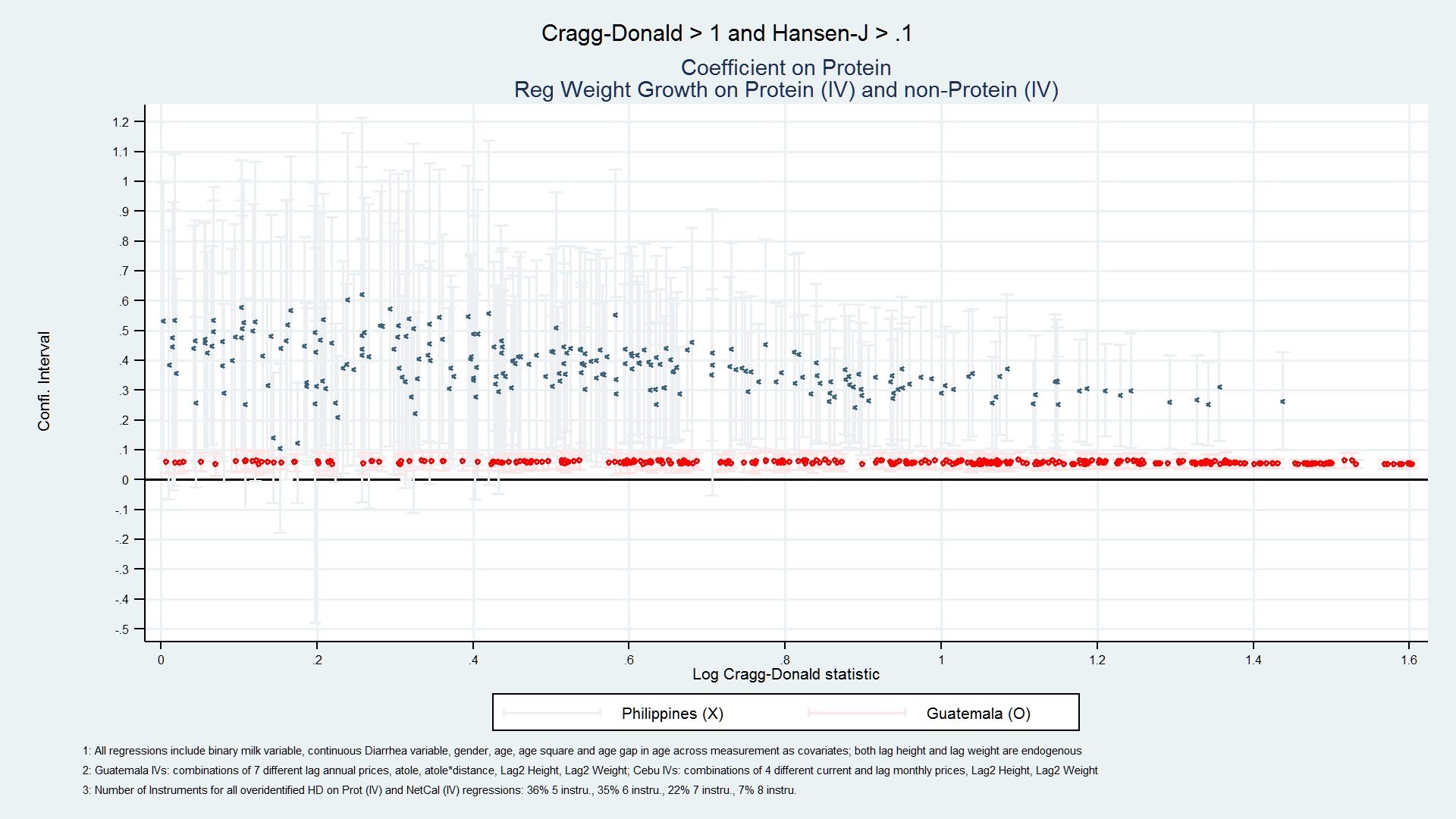


Figure A7: Non-Protein Coefficient

Change in Height: Estimates $\lambda_{non\_prot}^{h}$ from Equation (6) in the manuscript


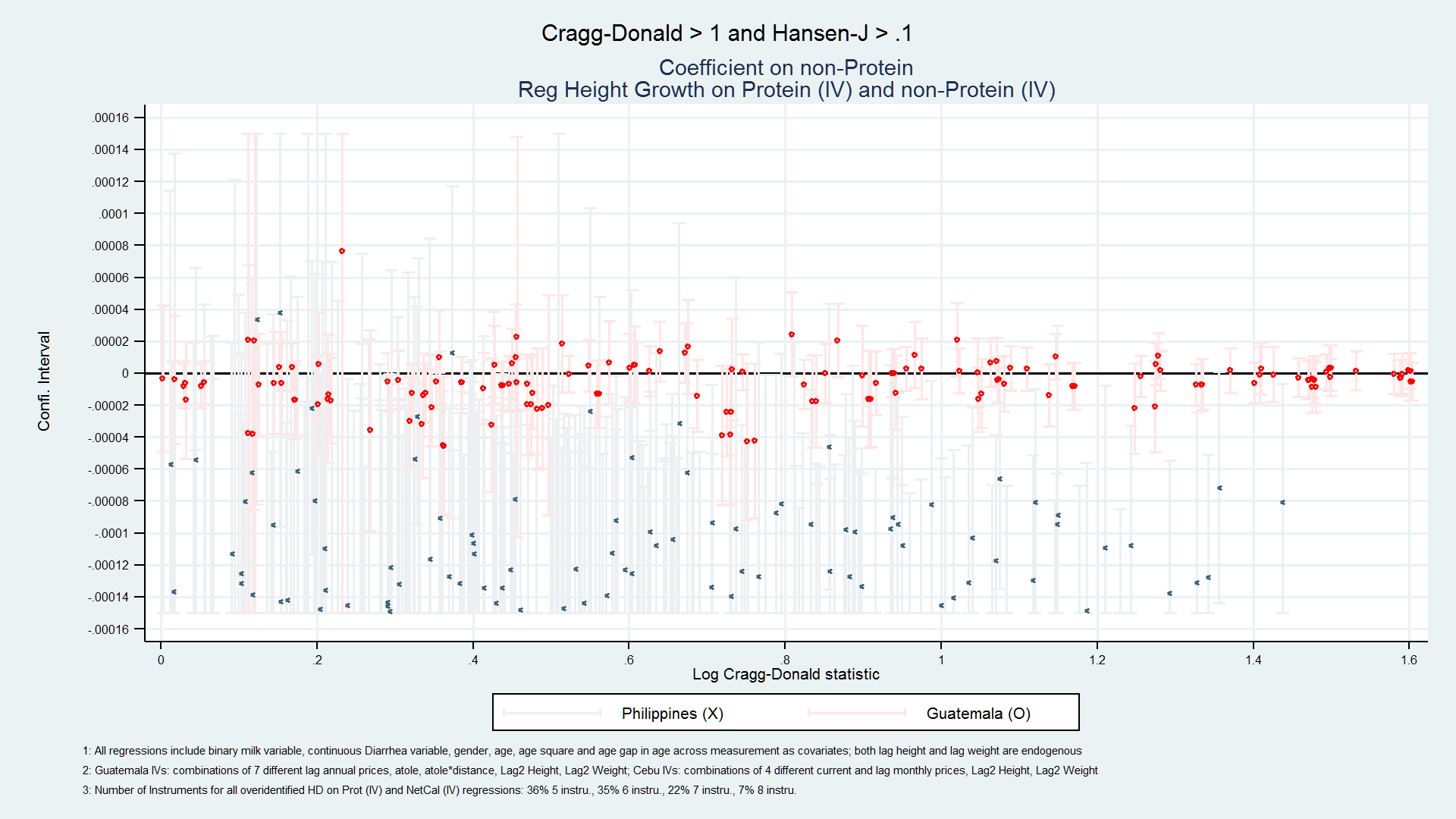


Change in Weight: Estimates $\lambda_{non\_prot}^{w}$ from Equation (6) in the manuscript


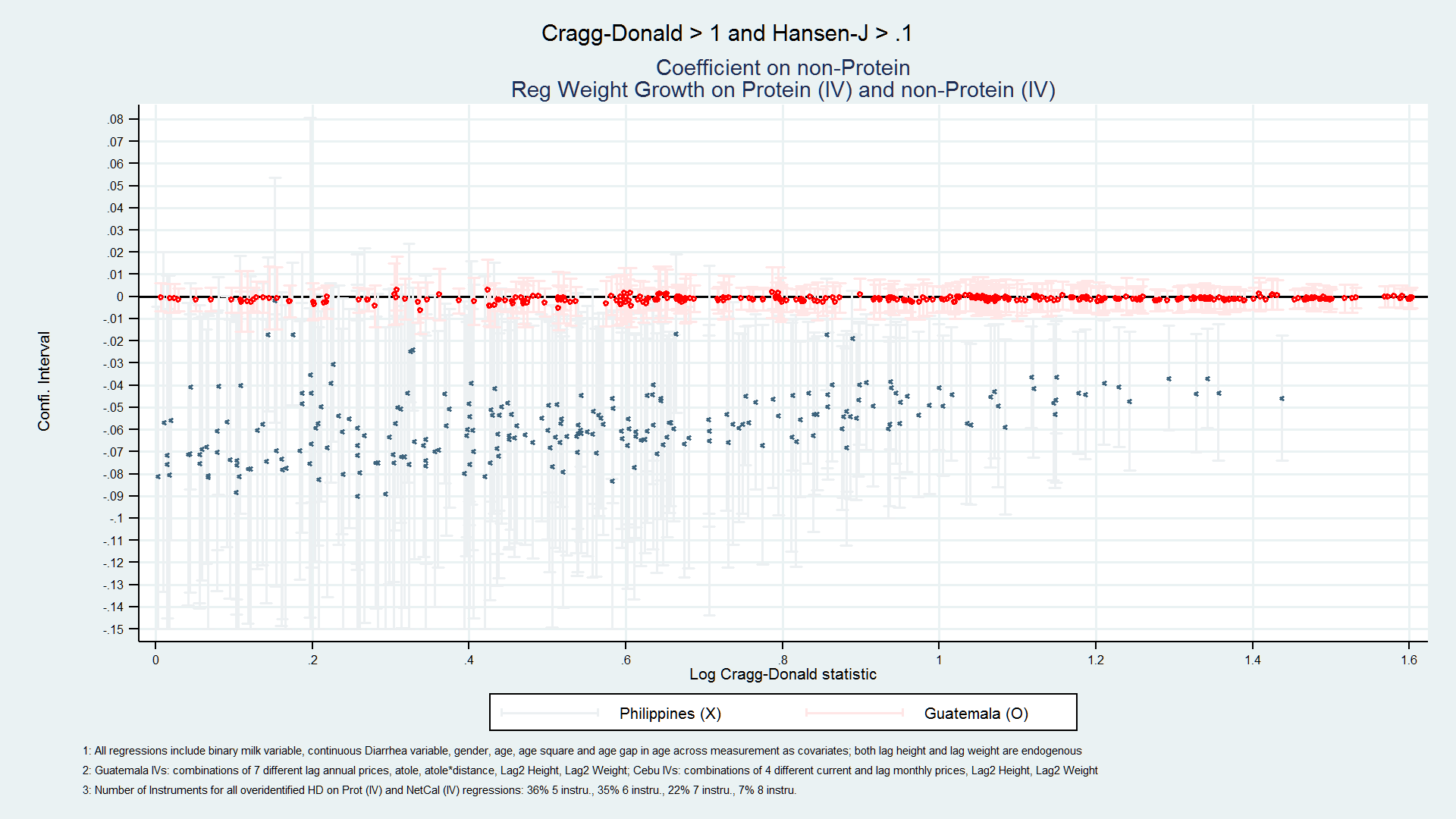


**6. Specifications used in the tables and graphs**

| Guatemala |
| --- |
| Potential Instruments: Atole, Distance multiplied with Atole and prices of eggs, chicken, pork, beef, rice, beans, and corn. |
| Specifications used for Guatemala follow the following criteria: |
|  |
| 1. Dependent variables: Change in Height and Change in Weight. |
| Atole and all combinations of 3 or 4 of the 7 prices |
| Atole, Distance multiplied with Atole and all combinations of 2, 3 or 4 of the 7 prices |
|  |
| 2. Dependent variable: Change in Weight. |
| Atole, second lag of height and all combinations of 2, 3 or 4 of the 7 prices |
| Atole, Distance multiplied with Atole, second lag of height and all combinations of 2, 3 or 4 of the 7 prices |
|  |
| 3. Dependent variable: Change in Height |
| Atole, Distance multiplied with Atole, second lag of weight and all combinations of 2, 3 or 4 of the 7 prices |
| Atole, second lag of weight and all combinations of 2, 3 or 4 of the 7 prices |
|  |
| 4. Dependent variables: Change in Height and Change in Weight |
| Atole,  second lag of height and second lag of weight and all combinations of 2, 3 or 4 of the 7 prices |
| Atole, Distance multiplied with Atole, second lag of height and second lag of weight and all combinations of 2, 3 or 4 of the 7 prices |
|  |
|  |
| Philippines |
| Potential Instruments: Second lag of height, second lag of height, current prices of tomatoes, corn, eggs, and dried fish, lagged prices of tomato, corn, egg and dried fish. |
| Specifications used for Philippines follow the following criteria: |
|  |
| 1. Dependent Variables: Change in Height and Change in Weight |
| All combinations of 4, 5 or 6 of the 8 prices |
|  |
| 2. Dependent variable: Change in weight |
| Second lag of height and all combinations of 3, 4, 5 or 6 of the 8 prices |
|  |
| 3. Dependent variable: Change in Height: |
| Second lag of weigh and all combinations of 3, 4, 5 or 6 of the 8 prices |
|  |
| 4. Dependent Variables: Change in Height and Change in Weight |
| Second lag of height and second lag of weight and all combinations of 2, 3, 4, 5 or 6 of the 8 prices |

1. This information was organized by Humberto Méndez at INCAP during June-September 2013. The information that he used had 15-day intervals. [↑](#footnote-ref-1)
2. Remember that the data available in the Philippines allow constructing only the number of days with diarrhea in the last 7 days prior to the height and weight measurements, but we need to construct estimates for a two-month period. [↑](#footnote-ref-3)
3. Communities correspond to what is known locally as the barangay—the smallest administrative division in the Philippines. [↑](#footnote-ref-4)
